# Pyroglutamyl aminopeptidase 1 is a potential molecular target toward diagnosing and treating inflammation

**DOI:** 10.3389/fimmu.2023.1301539

**Published:** 2023-11-30

**Authors:** Hongfei Jiang, Qiuyu Gong, Renshuai Zhang

**Affiliations:** ^1^ The Affiliated Hospital of Qingdao University, Qingdao University, Qingdao, China; ^2^ Department of Thoracic Surgery, The First Affiliated Hospital of Xi’an Jiaotong University, Xi’an, China

**Keywords:** pyroglutamyl aminopeptidase 1 (PGP-1), inflammation, biomarker, anti-inflammatory therapy, cancer

## Introduction

Inflammation is the body’s protective response to detrimental irritants (such as pathogens or irritants), which can clear out necrotic cells and initiate tissue repair. However, when inflammation becomes chronic or lasts too long, it can be harmful and may lead to a wide variety of diseases, including cardiovascular diseases, cancer, diabetes, arthritis, Alzheimer’s disease, pulmonary diseases, and autoimmune diseases (1). The COVID-19 that swept the world has further increased scientists’ attention to inflammation research. Many signaling pathways have been reported to be involved in the initiation and progression of inflammation, and some diagnosis markers for inflammation have been widely used in clinic, such as leukocyte count, procalcitonin, C-reactive protein, and IL-6. However, clinically available targets for inflammation diagnosis, especially treatment, are still relatively few. The discovery of novel inflammatory biomarkers has important implications for the diagnosis and treatment of inflammation and inflammation-related diseases. In recent years, the connection between pyroglutamyl aminopeptidase 1 (PGP-1, EC 3.4.19.3) and inflammation has received increasing attention from scientists. This viewpoint provides our opinions on PGP-1 as a promising novel target of inflammation.

## Evidence

**Figure 1:**
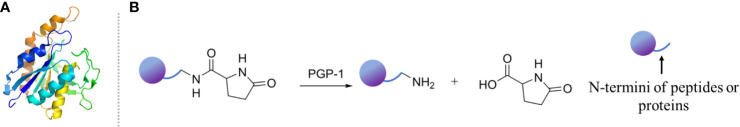
**(A)** Crystal structure of PGP-1 (PDB: 5Z48). **(B)** Catalytic hydrolysis mechanism of PGP-1.

PGP-1 is a kind of pyroglutamyl peptidase, which is a typical cysteine peptidase catalyzing the removal of L-pyroglutamate (pGlu) from the N-terminus of some peptides and proteins (**Figures 1A, B**) (2). PGP-1 has been found in almost all domains of life including mammals, avian, fish, plants, protists, and fungi. In addition, PGP-1 is highly conserved and ubiquitously distributed in human tissues, and its novel physiological roles are constantly being proposed in recent decades. In a previous study, PGP-1 was thought to be involved in the absorption of peptides and proteins from the mammalian alimentary tract due to its presence in both the small intestine and duodenum (3). In addition, also based on the widespread distribution of PGP-1 in functionally dissimilar tissues, researchers proposed that PGP-1 may participate in the intracellular catabolism and resynthesis of peptides (4). PGP-1 was also believed to affect certain disease states (*e.g.*, memory impairment) by regulating the levels of free pGlu, given the proven pharmacological properties of pGlu (5). Furthermore, PGP-1 exerted physiological roles by regulating physiologically important peptide hormones with pGlu at the N-terminus (*e.g.*, thyrotropin-releasing hormone and gonadotropin-releasing hormone) (6). Our group reported the first PGP-1 probe in 2016, and with help of the probe, we found that the cellular inflammation was accompanied by an increase of PGP-1 (7). Subsequently, we utilized a near-infrared PGP-1 fluorescent probe to further reveal the up-regulation of PGP-1 in an inflammatory mouse model (8). In addition, our study also suggested for the first time that the knocking down of PGP-1 led to the weakness of the inflammatory process (down-regulation of TNF-α expression) in RAW264.7 cells. Based on these studies, we proposed that PGP-1 was a new potential inflammatory cytokine.

In the following years, the relationship between PGP-1 and many inflammatory models was demonstrated utilizing various fluorescent probes. These inflammatory models included Freund’s incomplete adjuvant or lipopolysaccharide-induced macrophagocyte (RAW264.7), human normal hepatocyte cell (LO-2), hepatocellular carcinoma cell (HepG 2), human ovarian cancer cell (ES-2), and a mouse model. For example, Hu et al. designed a bioluminescent probe for quantitating PGP-1 activity by attaching a pGlu group onto aminoluciferin using an amide bond (9). The imaging study of living cells demonstrated the up-regulation of PGP-1 in the human ovarian cancer cell line ES-2 that was preincubated with a common immunopotentiator, Freund’s incomplete adjuvant. *In vivo* imaging also revealed that PGP-1 was highly expressed in a mouse model of inflammatory liver disease. It is worth noting that inflammation has been considered to be involved in the development and progression of tumors (10). The relationship between PGP-1 and inflammation-related diseases, especially tumors, has also been demonstrated in various tumor cell models (HepG2, Huh-7, A549, and HCT116) and tumor-bearing mice models (HepG2 tumor-bearing mice). Targeting inflammatory factors has been regarded as an important strategy for hepatocellular carcinoma (HCC) diagnosis, and treatment considering chronic inflammation is strongly associated with HCC. For example, the study from Guo et al. found that PGP-1 was aberrant expressed in HCC cell lines (HepG 2 and HuH-7) and HepG2 tumor-bearing mice (11). The overexpression of PGP-1 in HCC cells promoted tumor progression, whereas the knockdown of PGP-1 significantly suppressed tumor cell growth and migration. These data suggested that PGP-1 is involved in the development of HCC and can be regarded as a potential HCC biomarker and therapeutic target. In addition, Liu’s group also explored the expression of PGP-1 in HepG2, RAW264 cells, inflammation model mice pre-cultured with lipopolysaccharide, and HepG2 tumor-bearing mice by constructing ratiometric fluorescent sensors (12, 13). The imaging results of the inflammation model mice pre-cultured with lipopolysaccharide showed that the content of PGP-1 did increase after lipopolysaccharide induction. Furthermore, the *in vivo* imaging of HepG2 tumor-bearing mice also suggested that the expression level of PGP-1 in the living body was closely related to inflammation-related tumors. Moreover, the up-regulation of PGP-1 in burnt skin tissues and serum samples from burns patients during the occurrence of inflammation resulting from burns has also been demonstrated (14). These findings together suggest that PGP-1 is indeed involved in inflammatory response *in vitro* and *in vivo*.

## Discussion

PGP-1, as a novel inflammatory cytokine, has been demonstrated at the cellular level and in animal models and patient samples. However, more efforts are needed to identify GP-1 as a clinical indicator and therapeutic target for inflammation and inflammation-related diseases. Several problems deserve the attention of scientists.

Firstly, the direct mechanism that PGP-1 involves inflammation progress is complicated and unclear. Although the classical IL-6/STAT3 pathway is thought to play key roles in PGP-1 promoting the progression of hepatocellular tumor associated with chronic inflammation, how PGP-1 is involved in the occurrence and progression of inflammation still needs more direct evidence (11). Elucidating the reaction between PGP-1 and inflammation-related proteins may provide a solution to this issue since the dissociation of N-terminal peptide bonds is the typical functional feature of PGP-1.

Secondly, the feasibility of PGP-1 as a therapeutic target for inflammation has not yet been demonstrated *in vivo*. The development of PGP-1 inhibitors is key to solving this issue. As early as 1982, Fujiwara et al. reported a specific inhibitor for PGP-1, N-carbobenzoxypyroglutamyl diazomethyl ketone, which covalently bonds to the activity site with Ki of 0.12 mM (15). In addition, Ma group reported seven natural inhibitors against PGP-1, which showed inhibition activity against PGP-1 at micromolar level (16, 17). It is worth noting that, up to now, only a few PGP-1 inhibitors have been reported, and studies on their activity *in vivo* are lacking. Therefore, it would be extremely useful to develop effective PGP-1 inhibitors for exploring the role of PGP-1 as a therapeutic target for inflammation. On the one hand, the analysis and summary of the characteristics of PGP-1 substrates can provide a reference for the design of inhibitors. PGP-1 shows broad substrate specificity; however, it is highly specific for amino-terminal pGlu residues. Minor alterations to the pGlu residue may significantly affect the ability of PGP-1 to cleave the adjacent peptide bond. For example, increasing the size of the pyroglutamyl ring from 5 to 6 members or the introduction of a second ureido nitrogen into the ring almost completely eliminates the ability of PGP-1 to cleave the adjacent peptide bond (18). In addition, if the amino-terminal pGlu residues are attached to proline (Pro), the pGlu-Pro bonds are not normally hydrolyzed by mammalian PGP-1 (19). On the other hand, virtual screening is also an ideal strategy to obtain PGP-1 inhibitors. It is worth noting that the human PGP-1 enzyme has been purified and enzymatically characterized; however, its crystal structure is not known (20). Virtual screening based on non-human species PGP-1 has limited accuracy. It is believed that with the elucidation of the human PGP-1 structure, virtual screening will greatly improve the probability of PGP-1 inhibitor discovery.

Thirdly, it has been widely recognized that chronic inflammation might result in carcinogenesis, and this induced process usually goes on silently for a long time (10). PGP-1 inhibitors have the potential to prevent tumorigenesis and progression by inhibiting chronic inflammation. If the PGP-1 inhibitors used in anti-chronic inflammation are derived from food materials, they may be easier to incorporate into the daily diet and easier to use for cancer prevention. Thus, inhibitors of PGP-1 derived from food materials, such as edible plants, should be of greater concern in the prevention of inflammation-related tumors.

In conclusion, PGP-1 shows great potential in the diagnosis and treatment of inflammation and inflammation-related diseases. However, more investigation to elucidate the detailed mechanism of PGP-1’s involvement in inflammation progression and to further validate the effectiveness of PGP-1 inhibitors *in vivo* is still needed.
