# Corrigendum: Pyroglutamyl aminopeptidase 1 is a potential molecular target toward diagnosing and treating inflammation

**DOI:** 10.3389/fimmu.2024.1538819

**Published:** 2024-12-19

**Authors:** Hongfei Jiang, Qiuyu Gong, Renshuai Zhang

**Affiliations:** ^1^ The Affiliated Hospital of Qingdao University, Qingdao University, Qingdao, China; ^2^ Department of Thoracic Surgery, The First Affiliated Hospital of Xi’an Jiaotong University, Xi’an, China

**Keywords:** pyroglutamyl aminopeptidase 1 (PGP-1), inflammation, biomarker, anti-inflammatory therapy, cancer

In the published article, there was an error in the **Funding** statement. The **Funding** statement for the Natural Science Foundation of Shandong Province (ZR2021QH212) is wrong. The correct statement is “Shandong Postdoctoral Science Foundation (SDCX-ZG-202203015). The correct **Funding** statement appears below.

“The author(s) declare financial support was received for the research, authorship, and/or publication of this article. This research received funding supporting from the Shandong Postdoctoral Science Foundation (SDCX-ZG-202203015) and the Key Research and Development Project of Shaanxi Province (2023-YBSF-292).”

The authors apologize for this error and state that this does not change the scientific conclusions of the article in any way. The original article has been updated.

